# Evaluation of left atrial function and the relationship between left atrial stiffness index and exercise capacity in hypertension-related heart failure with preserved ejection fraction

**DOI:** 10.3389/fcvm.2024.1501004

**Published:** 2024-12-17

**Authors:** Qingfeng Zhang, Sijia Wang, Hongmei Zhang, Kai Wang, Wenhua Li, Geqi Ding, Luwei Ye, Chunmei Li, Yan Deng, Yi Wang, Lixue Yin

**Affiliations:** ^1^ School of Medicine, University of Electronic Science and Technology of China, Chengdu, China; ^2^ Department of Cardiovascular Ultrasound and Key Laboratory in Cardiac Electrophysiology and Biomechanics, Sichuan Provincial People’s Hospital, University of Electronic Science and Technology of China, Chengdu, China; ^3^ Department of Acute Care Surgery, Sichuan Provincial People’s Hospital, University of Electronic Science and Technology of China, Chengdu, China

**Keywords:** three-dimensional speckle tracking, left atrial, hypertension, stiffness, heart failure

## Abstract

**Objective:**

The left atrial stiffness index (LASI) holds significance in the atrioventricular coupling function and heart failure progression. To assess left atrial function and evaluate the relationship between LASI and exercise capacity in hypertension-related heart failure with preserved ejection fraction (HT-HFpEF).

**Methods:**

The study involved 62 healthy subjects and 163 patients with HT (112 patients in simple HT group and 51 patients in HT-HFpEF group). Each patient performed exercise stress test and standard ultrasonic images were evaluated. A comprehensive evaluation of atrioventricular function, along with investigation into the correlation between these functional parameters and exercise capacity. And further to investigate the feasibility of predicting exercise intolerance using three-dimensional derived left atrial strain index (LASI) (E/e'/LASr and E/e'/LASr-c).

**Results:**

Compared to healthy subjects, HT group demonstrated the elevation in left atrial volume accompanied by decrease in strain value (*P* < 0.05). In HT-HFpEF group, further significant reductions were observed in both longitudinal (LASr) and circumferential strain (LASr-c, LASct-c) (*P* < 0.05). Univariate regression demonstrated that both E/e'/LASr and E/e'/LASr-c were significantly correlated with metabolic equivalents (METs) (r = −0.462, *P* < 0.001; r = −0.381, *P* < 0.001). The E/e'/LASr demonstrates comparable diagnostic efficacy to exercise-E/e' in assessing exercise intolerance in HT-HFpEF patients (AUC: 0.836 vs. 0.867, *P* = 0.239).

**Conclusion:**

Progressive LA remodeling contributes to decreased atrioventricular compliance in HT and HT-HFpEF patients.E/e'/LASr serves as an independent indicator of exercise intolerance in patients with HT-HFpEF.

## Graphical Abstract

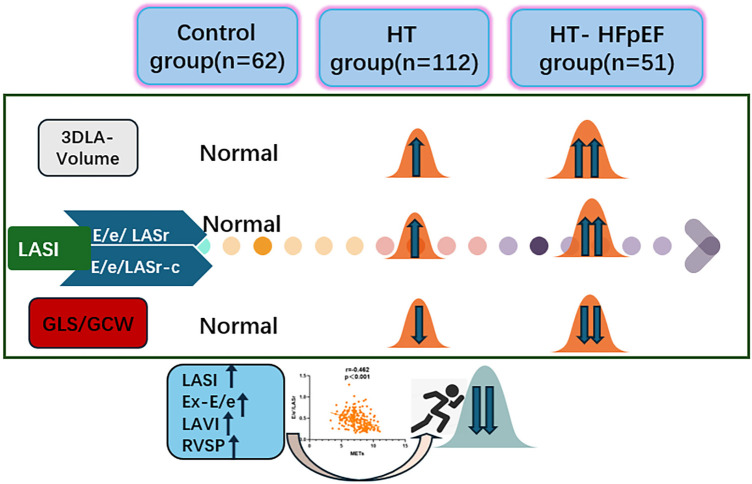

## Introduction

Heart failure with preserved ejection fraction (HFpEF) encompasses diverse etiologies and phenotypes, with hypertension (HT) accounting for 60%–70% of cases (1), this underscoring the need for more targeted studies on this cohort. The United States with experts supporting the affirmation that asymptomatic patients with hypertension should be classified as stage A Heart Failure (2). The underlying mechanisms by which atrioventricular function affects exercise tolerance in hypertension-related heart failure with preserved ejection fraction (HT-HFpEF) patients remain elusive.The emergence of three-dimensional speckle tracking imaging (3DSTI) technology has significantly improved reproducibility and accuracy in related assessment (3), potentially providing deeper insights into the pathophysiology underlying exercise intolerance (4). Additionally, metabolic equivalents (METs) is a standard unit used to measure the intensity of physical activity, and have been established as robust predictor of cardiovascular unfavorable outcomes (5). We hypothesized that three-dimensional left atrial(LA) speckle tracking can effectively assess atrioventricular compliance function in HT-HFpEF patients,and left atrial strain index (LASI) indices are independently associated with exercise capacity,may serve as effective tools for predicting exercise intolerance, particularly in HT-HFpEF patients.

## Methods

### Study subjects

**Figure 1:**
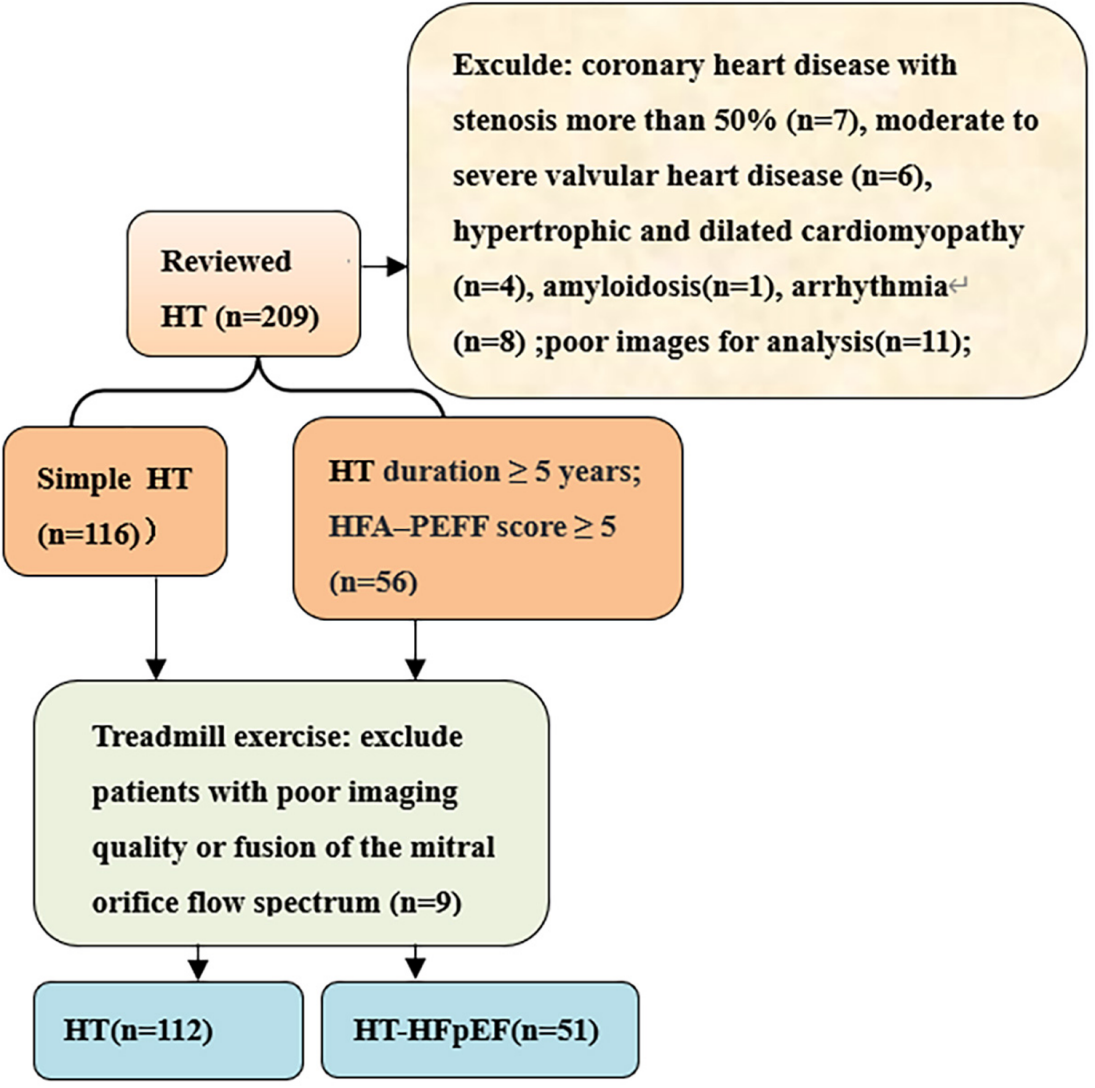
Flow diagram of participants selection.

We conducted prospective cross-sectional study on hypertensive patients who received medical care at the hospital from March 2022 to October 2023. The inclusion criteria for patients diagnosed with hypertension (HT) adhered strictly to the European Guidelines for the Prevention and Treatment of Hypertension (6), included 112 patients in HT group and 51 patients in HT-HFpEF group. The HT-HFpEF patients needed to satisfy the following criteria: duration of hypertension ≥5 years and according to the 2019 HFA–PEFF diagnostic algorithm: the consensus recommendation from the Heart Failure Association (HFA) of the European Society of Cardiology (ESC) (7), the score ≥ 5 points implies definite HFpEF; while mean score for the HT group was 2.9 ± 1.6. Patients with poor image quality, atrial fibrillation, moderate to severe valvular heart disease,congenital heart disease, hypertrophic and dilated cardiomyopathy,amyloidosis,coronary heart disease with stenosis more than 50%,constrictive pericarditis were excluded (Figure 1). We also included 62 normal subjects who underwent echocardiography examination during the same period with mean age of 56 ± 6 years.

### Transthoracic echocardiography

M5S and 4V1 probes of GE-vivid 95 instrument (GE Ultrasound, Horten, Norway) were utilized for the image acquisition. Patients were instructed to hold their breath for 5–7 s and the 3D image frame rate was ensured to exceed 25 frames per second. Left ventricular end-diastolic diameter (LVEDd), right ventricular systolic pressure(RVSP), left ventricular mass index(LVMI), biplane-derived ejection fraction(EF), stroke volume (SV), cardiac index (CI) were obtained. From apical four-chamber views, E and A wave values were measured by transmitral flow velocity, early diastolic (e') and late diastolic (a') velocity were obtained using tissue doppler imaging (TDI),thus allowing to calculate E/e’ ratio. Auto function imaging (AFI) was applied to obtain global longitudinal strain (GLS),Global work index (GWI) represents the total workload under the pressure-strain curve, which is the product of strain and systolic pressure. Global constructive work (GCW) contributes to LV ejection and is the sum of work performed during LV systolic myocardial shortening and isovolumic diastolic myocardial lengthening.Global wasted work (GWW) is the work performed by myocardial lengthening during systole or shortening during isovolumic relaxation. Global work efficiency (GWE) is calculated as the ratio of GCW/(GCW + GWW) (8).

The treadmill exercise stress test was conducted according to the Bruce protocol and the test was stopped once the target heart rate (220-age) reached 85% (9). We recorded hemodynamic parameters as peak systolic arterial pressure (SBP-peak), peak heart rate(HR-peak), METs, and echocardiography performed immediately after exercise to measure E/e'(Ex-E/e'). Effective arterial elastance (Ea) was calculated as end-systolic pressure(ESP)/SV,and LV end systolic elastance (Ees) was derived as ESP **{**end-systolic volume (ESV) -V0}, with V0 considered negligible compared to ESV. The Ea/Ees ratio, equivalent to ESV/SV,was used to determine ventricular-arterial coupling (10). Additionally, the total systemic vascular resistance index (SVRI) was determined using the formula{ (mean BP- right atrial pressure(RAP)} × 80/cardiac index (CI) (11), which reflects cardiac afterload,the estimation of RAP is based on the inferior vena cava diameter and its collapsibility with respiration (12).

### LA volume and strain analysis

LA images were analyzed using EchoPAC 204 workstation software(4D-auto LAQ),a well-established and widely applied technique (13). Manual adjustments were made on apical four-chamber, three-chamber, and two-chamber views at end-systole and end-diastole to outline the LA border while excluding pulmonary veins and LA appendage regions, the process performed by experienced sonographers. This process generated time-volume curve for LA, and provided strain data, including longitudinal and circumferential reservoir phase strain (LASr,LASr-c), conduit phase strain (LAScd,LAScd-c), pump phase strain (LASct,LASct-c),maximum LA volume (LAVmax),minimum LA volume (LAVmin), pre-atrial contraction LA volume (LAVpreA), LA ejection volume (LAEV) and LA emptying fraction (LAEF) were automatically calculated by 3D LA analysis (14). The left atrial volume index (LAVI) was calculated by normalizing LAVmax according to body surface area (BSA),the left atrial stiffness index-LASI (E/e'/LASr, E/e'/LASr-c) was evaluated by computing the ratio of E/e' value to LA reservoir strain.We referred to previous study by snader et al. (15), who studied exercise tolerance METs to predict all-cause mortality in population of 3,400 patients; METs <6 were considered to be associated with reduced and poor long-term prognosis. Therefore, we evaluated the diagnostic value of LASI, Ex-E/e', LAVI, and RVSP in HT-HFpEF patients with METs <6,and conducted Delong test.

### Statistical analysis

Statistical analysis was conducted using SPSS version 26.0.Continuous variables were compared using one-way analysis of variance (ANOVA) with Bonferroni correction or the non-parametric Kruskal-Wallis test. General linear regression analysis was conducted to evaluate the contributions of clinical variables, echocardiographic parameters, and exercise hemodynamic measures to exercise capacity. Variables identified as significant in univariable analysis were subsequently incorporated into multivariable models to determine independent predictors. An optimized model was generated, and partial r was defined as the association between the predicted variable and achieved METs. Key parameters were utilized for receiver operating characteristic (ROC) and area under the curve (AUC) analysis, to identify HT-HFpEF patients with reduced exercise capacity,and AUC values were compared using the DeLong test. Intra/inter-observer variability for LA function measurements was determined by estimating intraclass correlation coefficients (ICCs) for LA longitudinal strain indices including reservoir strain, conduit strain, and contraction strain, with ICCs of 0.92(95%CI: 0.82–0.97)/0.84(95%CI:0.68–0.93); 0.91(95%CI:0.81–0.94)/0.79(95%CI:0.61–0.92), and 0.89(95%CI:0.74–0.95)/0.81(95%CI:0.69–0.94), respectively.

## Results

### Demographics, clinical features, and echocardiographic parameters

Significant differences were identified in age, body mass index (BMI), and BSA among the three groups (*P* < 0.05). Additionally, variations in clinical medication were observed between different HT groups, as shown in Table 1. Significant differences were found among the three groups for LVEDd, LVMI, E/e' and RVSP (*P* *<* 0.05). Similarly, SV and CI showed significant differences across the groups (*P* < 0.05), except for EF (*P* *>* 0.05). Baseline GLS levels were notably lower in both the HT-HFpEF and HT groups compared to the control group (*P* < 0.05) (Table 2).

**Table 1:** Demographics and clinical features of the study population.

Variables	Control group (*n*=62)	HT group (*n*=112)	HT-HFpEF group (*n*=51)	*P*-value
Sex	36 (58.1)	77 (68.7)	30 (58.8)	0.272
Ag	56 ± 6	58 ± 7^a^	65 ± 6^a^^b^	<0.001
BSA (m^2^)	1.66 ± 0.12	1.74 ± 0.18^a^	1.76 ± 0.15^a^	0.006
BMI	21.9 (20.7,23.5)	22.95 (21.5,24.2)^a^	25.3 (23.5,26.7)^a^^b^	<0.001
Dyslipidemia (%)	2	32 (28.5)^a^	21 (41)^a^^b^	<0.001
DM	/	9 (6.2)	6 (9.8)	0.16
Medication				
Beta blocker (%)	/	32 (28.6)	13 (25.4)	0.42
ACEI or ARB	/	58 (51.7)	29 (56.8)	0.61
Statin (%)	/	34 (30.3)	28 (54.9)	0.003
Aldosterone inhibitors (%)	/	14 (12.5)	9 (17.6)	0.17
Diuretics	/	15 (13.4)	14 (27.5)	0.007
NT-proBNP	19.6 (15.9, 27.1)	89.1 (63.7,114.9)^a^	187 (148.5,266.4)^a^^b^	<0.001

HT, Hypertension; HFpEF, heart failure with preserved ejection fraction; ACEI, angiotensin-converting enzyme inhibitor; ARB, angiotensin receptor blocker; BSA, body surface area; BMI, body mass index; DM, diabetes dellitus; NT-proBNP, N-terminal pro-B-type natriuretic peptide.

^a^ refers to comparison with the control group.

^b^ refers to comparison with the HT group.

**Table 2:** Comparison of echocardiographic and exercise parameters.

Variables	Control group (*n*=62)	HT group (*n*=112)	HT-HFpEF group (*n*=51)	*P*-value
General echo parameters				
HR-rest	76.1 ± 9.2	69.2 ± 9.8^a^	68.1 ± 9.0^a^	<0.001
SBP-rest (mmHg)	121.1 ± 10.2	139.5 ± 14.7^a^	142.1 ± 13.1^a^	<0.001
E (m/s)	0.87 (0.72,1.04)	0.83 (0.7,0.97)	0.80 (0.71,0.96)	0.255
A (m/s)	0.87 ± 0.2	0.91 ± 0.21	0.92 ± 0.17	0.163
E/e’	6.7 (6.1,7.7)	8.7 (6.9,10)^a^	10.6 (9.8,11.4)^a^^b^	<0.001
LVEDd (mm)	45.8 ± 3.1	47.6 ± 3.7^a^	48.9 ± 3.5^a^^b^	0.009
LVMI (g/m^2^)	98.5 (96,105.8)	117.1 (106.4,123.1)^a^	119 (110.2,125.9)^a^	<0.001
RVSP (mmHg)	25.5 (22.4,27.1)	28.7 (26.5,30.5)^a^	32.4 (28.8,35.5)^a^^b^	<0.001
EF	0.68 ± 0.06	0.67 ± 0.05	0.65 ± 0.05^a^	0.48
SV(ml)	52 (47,56)	51 (46,57)	48 (46,54)^a^	0.041
CI(l/min/m^2^)	2.9 ± 0.7	2.63 ± 0.5^a^	3.0 ± 0.6^b^	0.001
GLS(%)	21.6 (20.4,22.4)	19.5 (17.2,21.8)^a^	17.8 (16.5,19.6)^a^^b^	<0.001
Myocardial work				
GWI (mmHg%)	2018.5 (1966,2191.5)	2121.5 (1860.2,2247)^a^	1890 (1743.5,1992.5)^a^^b^	<0.001
GCW (mmHg%)	2412.5 (2270.5, 2521.7)	2540 (2116.7,2595.5)^a^	2210 (1980,2310.5)^a^^b^	<0.001
GWW(mmHg%)	42 (28.3,58.3)	59 (38.7,93.5)	89 (57.5,119)^a^^b^	<0.001
GWE	0.98 (0.97,0.98)	0.97 (0.95,0.98)^a^	0.93 (0.9,0.95)^a^^b^	<0.001
Ventricular-arterial coupling				
Ea/Ees	0.48 (0.43,0.64)	0.53 (0.47,0.63)	0.52 (0.42,0.6)	0.109
SVRI (dyn·s/cm^5^/m^2^)	2021.1 (1918.6,2344)	2566.7 (2108.8,2942.3)^a^	2796.6 (2394.3,3312.1)^a^^b^	<0.001
Exercise parameters				
SBP-peak (mmHg)	160.2 (146,181)	166 (156.3,189)^a^	176 (158,205)^a^	<0.001
HR-peak	150.6 ± 16.8	148.1 ± 15.6	143.9 ± 12.9	0.477
METs	8.9 ± 0.9	7.2 ± 1.4^a^	6.4 ± 0.9^a^^b^	<0.001
Ex-E/e’	6.5 (5.5,7.5)	10.65 (8.7,12.3)^a^	14.6 (13.7,15.5)^a^^b^	<0.001

HT, hypertension; HFpEF, heart failure with preserved ejection fraction; LVEDd, left ventricular end-diastolic diameter; E, peak velocity of mitral early diastolic flow; A, late diastolic flow; e’, early diastolic mean velocity of mitral annulus; RVSP, right ventricular systolic pressure; EF, ejection fraction; SV, stroke volume; CI, cardiac index; GLS, global longitudinal strain; METs, metabolic equivalents; Ex-E/e’, exercise E/e’; GWI, global work index; GCW, global constructive work; GWW, global wasted work; GWE, global work efficiency, LV end systolic elastance (Ees),effective arterial elastance(Ea),Ea/Ees=end-systolic volume(ESV)/SV; systemic vascular resistance index(SVRI),SVRI={(mBP-RAP)×80}/CI.

^a^ indicates comparison with the control group.

^b^ indicates comparison with the HT group.

### Myocardial work and ventricular-arterial coupling

Myocardial work indices, which serve as load-independent parameters revealed decrease in GWI, GCW, and GWE in HT-HEpEF group (*P* < 0.05). Conversely, in the HT group, GWI/GCW exhibited a significant increase (*P* < 0.05), which is consistent with previous findings (16). Ea/Ees as metric indicative of ventricular-arterial coupling, did not vary significantly (*P* > 0.05); However, SVRI was significantly elevated in HT-HFpEF patients compared to those in HT group, with the lowest values in normal subjects. (*P* < 0.05). In terms of post-exercise variables, HR-peak did not differ significantly between the groups (*P* > 0.05). However, as expected, Ex-E/e' was significantly higher in HT-HFpEF group, and METs reflect exercise capacity was markedly lower compared to the other two groups (*P* < 0.05) (Table 2).

### LA volume and strain analysis

As depicted in Table 4, the values of LAVmin, LAVmax, LAVI, and LAEF were significantly elevated in HT group (*P* < 0.05). In the HT-HFpEF cohort, LA volume variables were increased at all time phases; however, for LAEF, longitudinal and circumferential strain were notable decreased (*P* < 0.05).LASr, LASr-c, LASct-c were differed significantly between the HT and HT-HFpEF groups (*P* < 0.05), thus indicating that these represent may serve as sensitive indicators of cardiac dysfunction in HT-HFpEF patients.

**Figure 2:**
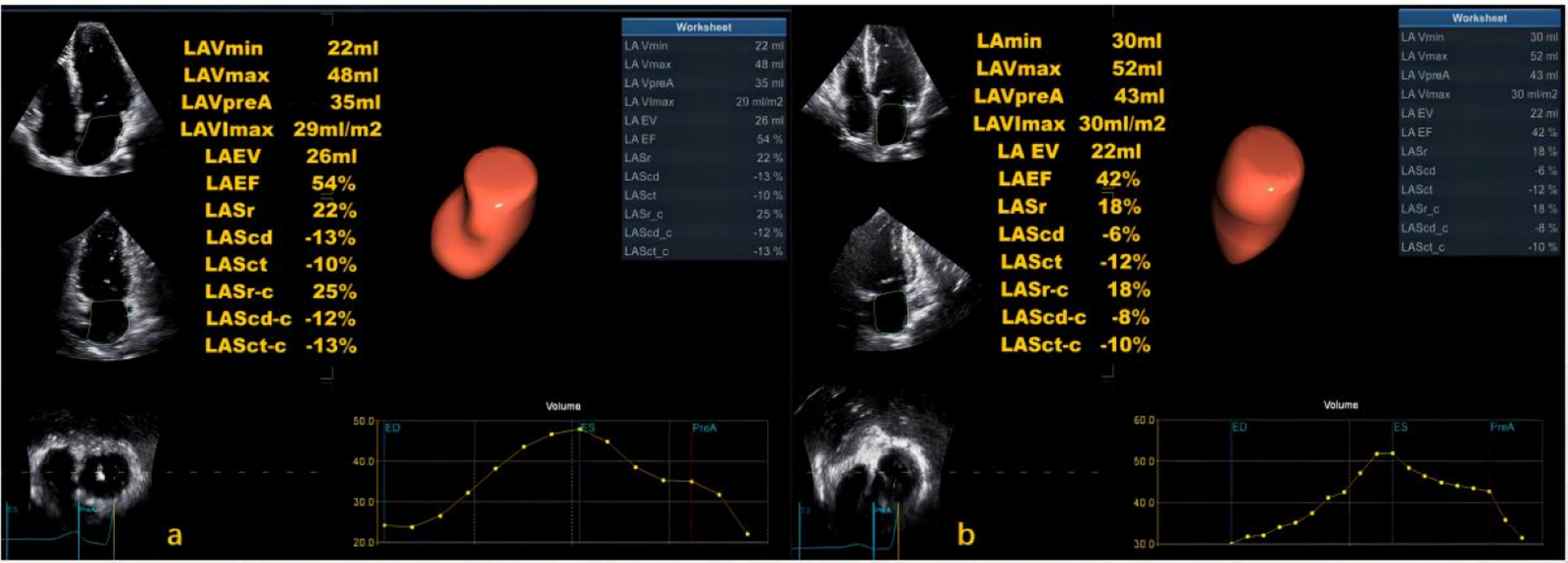
Three-dimensional (3D) left atrial volume and strain analysis for a control patient **(a)** and HT-HFpEF patient **(b).**

**Figure 3:**
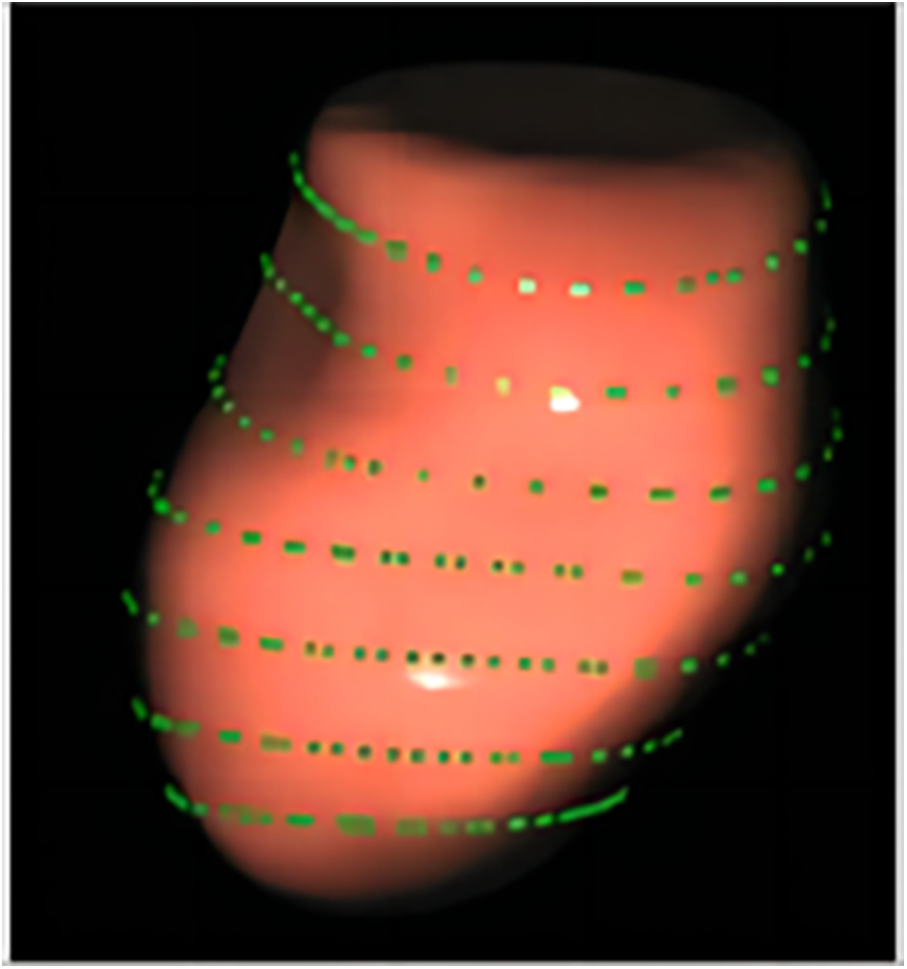
The points used to determine three-dimensional (3D) circumferential lines for the circumferential strain calculation.

The LASI, derived from left atrial strain, showed a consistent upward trend, being significantly higher in the HT-HFpEF group (*P* < 0.05),specifically, the ratio of E/e' to LASr was 0.58 (0.52, 0.66) in the HT-HFpEF group vs. 0.46 (0.33, 0.59) in the HT group, similarly, the ratio of E/e' to LASr-c was 0.5 (0.38, 0.6) vs. 0.36 (0.27, 0.43). These findings emphasize LASI as key index for evaluating impaired atrioventricular compliance in HT-HFpEF patients, underscoring its critical role in detecting atrioventricular dysfunction in this population (Table 3). Conversely, no significant differences were observed in LAScd and LAScd-c between the HT and HT-HFpEF groups, indicating that conduction function remains preserved (*P* > 0.05). Figure 2 presents an example of different time-phase 3D LA volume and strain parameters for HT-HFpEF and control patient. Figure 3 depicts the points utilized for determining circumferential strain lines.

**Table 3:** Comparison of LA volume and strain parameters.

Variables	Control group (*n*=62)	HT group (*n*=112)	HT-HFpEF group(*n*=51)	*P*-value
LA volume				
LAVmin	19 (16,22.8)	27 (20,33.2)^a^	31 (25,34)^a^^b^	<0.001
LAVmax	44.7 ± 11.7	52.1 ± 12.3^a^	55.2 ± 9.0^a^	0.001
LAVpreA	31.3 ± 7.1	39.8 ± 9.1^a^	39.1 ± 8.7^a^	<0.001
LAVI	24.7 ± 4.7	30.1 ± 6.2^a^	32.7 ± 4.2^a^^b^	<0.001
LAEV	22.5 (20,26)	25 (22,30)^a^	24.3 (21,28.5)	0.029
LAEF	55.3 ± 5.3	49.1 ± 6.5^a^	45.5 ± 6.6^a^^b^	<0.001
LA strain				
LASr	26 (22.3, 28.7)	23 (17.8,27)^a^	17 (16.5,21)^a^^b^	<0.001
LAScd	13 (11,15.7)	11 (7,12)^a^	10 (8,12)^a^	<0.001
LASct	13.9 ± 4.3	9.9 ± 3.9^a^	9.8 ± 3.0^a^	<0.001
LASr-c	30.1 ± 5.6	25.6 ± 6.5^a^	22.4 ± 6.4^a^^b^	<0.001
LAScd-c	14 (11,16.7)	11 (8,14)^a^	11 (9,13)^a^^b^	<0.001
LASct-c	17.3 ± 3.8	15.38 ± 6.0^a^	13.5 ± 5.7^a^	0.087
LA stiffness index				
E/e’/LASr	0.27 (0.22,0.33)	0.46 (0.33,0.59)^a^	0.58 (0.52,0.66)^a^^b^	<0.001
E/e’/LASr-c	0.23 (0.19,0.27)	0.36 (0.27,0.43)^a^	0.5 (0.38,0.6)^a^^b^	<0.001

HT, hypertension; HFpEF, heart failure with preserved ejection fraction; LASr,LASr-c, longitudinal and circumferential reservoir phase strain; LAScd,LAScd-c, conduit phase strain; LASct,LASct-c, pump phase strain; LAVmax, maximum LA volume; LAVmin, minimum LA volume; LAVpreA, pre-atrial contraction LA volume; LAEV, LA ejection volume; LAEF, LA emptying fraction; LAVI, left atrial volume index.

^a^ indicates comparison with the control group.

^b^ indicates comparison with the HT group*.*

### Univariate and multivariate regression: independent associations of exercise capacity

**Figure 4:**
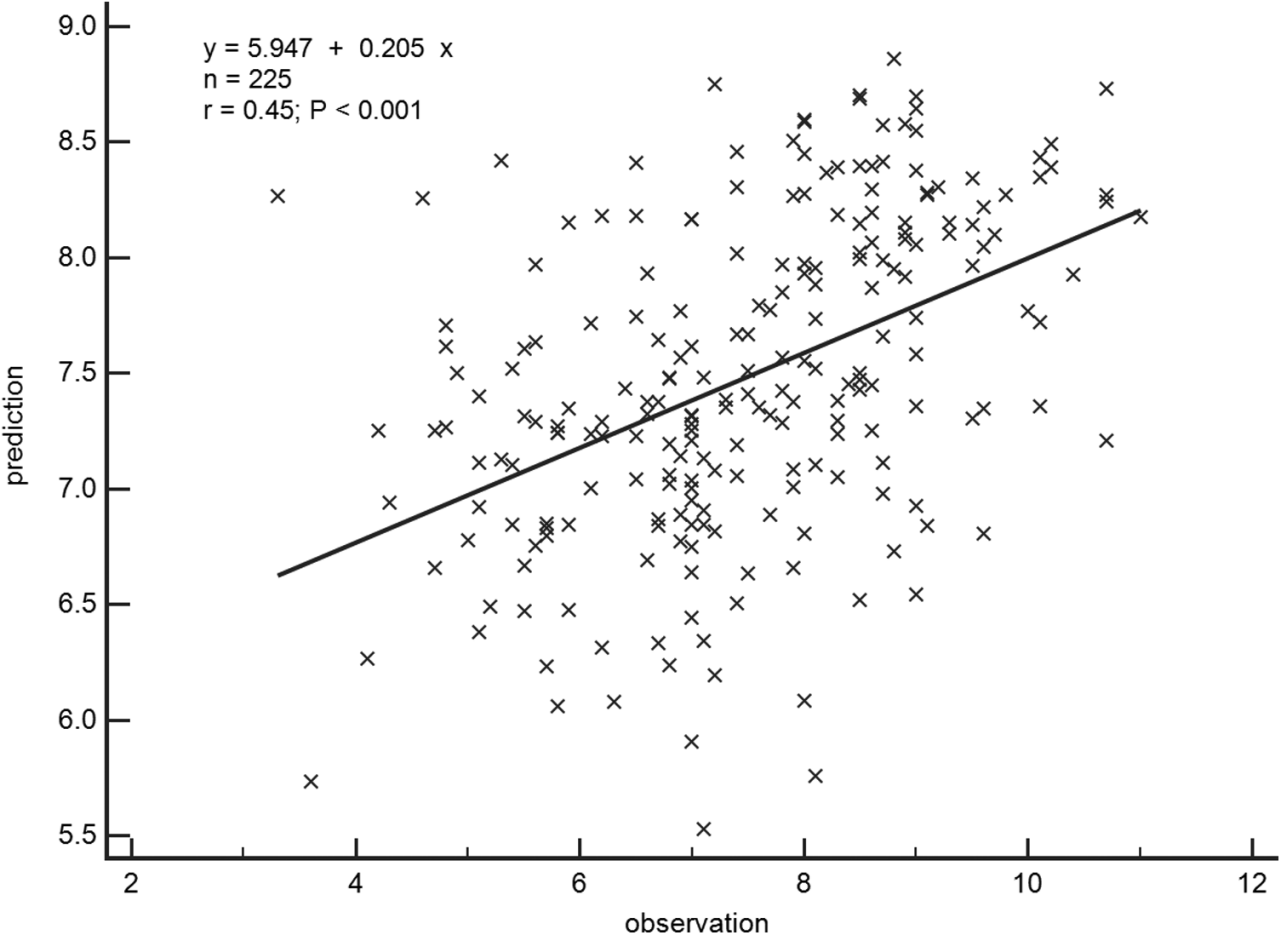
Overlay scatter plots of the METs achieved in comparison with the METs predicted. Partial r represented the strength of association between the variable and METs achieved, based on the predicted values derived from Model 3.

**Figure 5:**
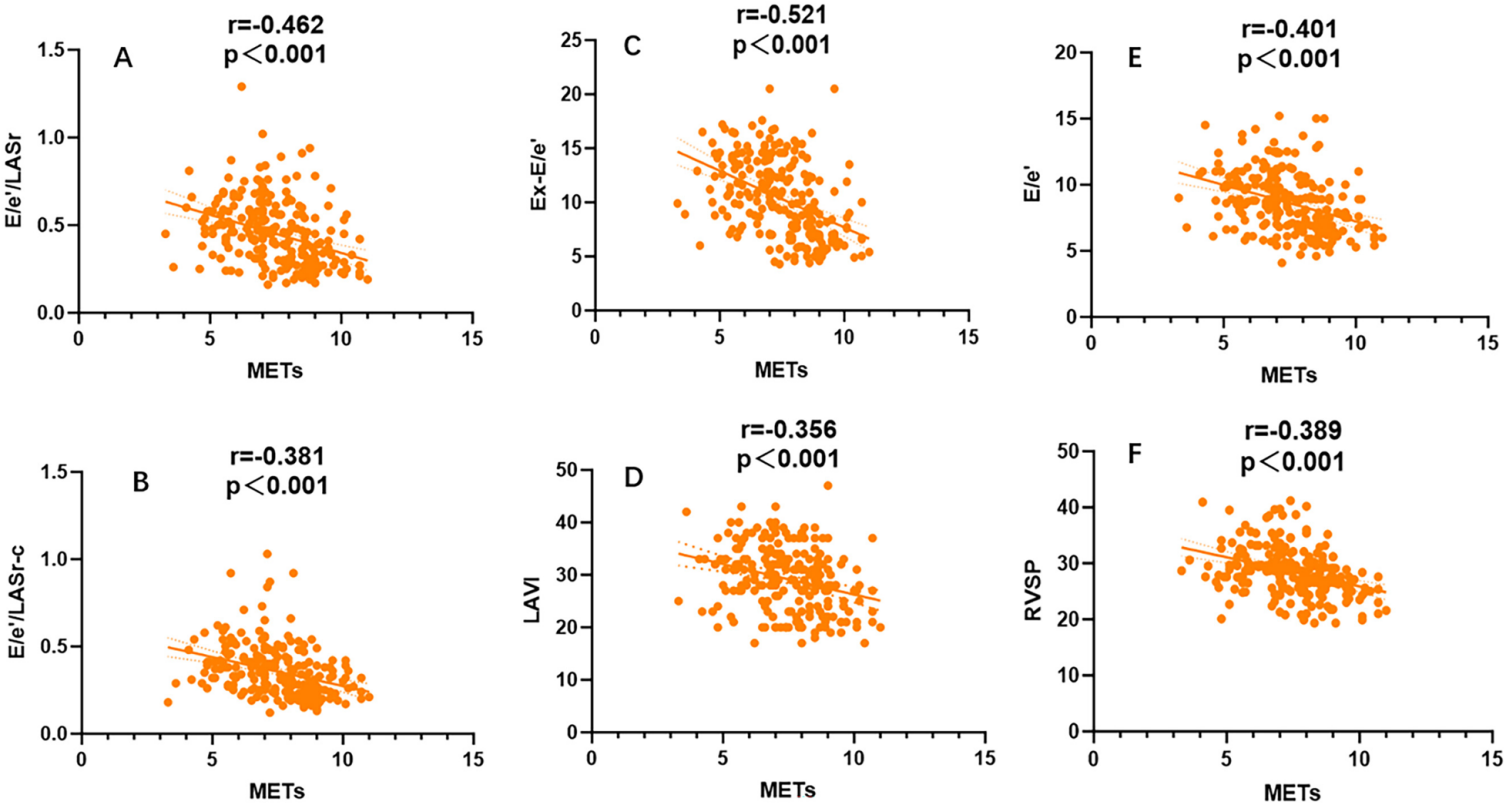
Univariate correlation analysis for LASI, LAVI,Ex-E/e’,RVSP,E/e’ coefficient with METs, All combinations were significantly correlated (*P* < 0.001).

A general linear regression analysis was conducted to assess the relative contribution of each variable to exercise capacity through a heatmap,and lambda plots was used to facilitate analysis. Significant statistical correlations (*P* < 0.05) were observed between LA function (inclusive of volume and strain), GLS, GCW, SVRI and METs; indicated that these parameters could be considered as candidate variables in multiple regression models to determine the independent predictors of METs. Subsequently, Age, SBP-rest, and BMI emerged as independent general predictors of METs. And an analysis combining E/e'/LASr, E/e'/LASr-c with LVAI, GLS, E/e', RVSP and SVRI was conducted (referred as echocardiographic Model 2). Ultimately, a comprehensive model that integrates both general and echocardiographic predictors (Model 3) and found that E/e'/LASr still independently related, in Table 4. To assess the clinical value of E/e'/LASr, we utilized unstandardized coefficients from Model 3 to generate overlay scatter plots, comparing METs achieved during exercise with those predicted by the model, the results demonstrated strong positive linear correlation. Partial *r* represented the strength of association between the model variable and METs achieved (Figure 4). Notably, LASI represents the elastic modulus during diastole, and serves as a marker of myocardial stiffness or flexibility. The correlation diagram between LASI (E/e'/LASr, E/e'/LASr-c),candidate variables and METs are displayed (*P* < 0.05) (Figure 5). The strongest correlations were observed between Ex-E/e’ and METs (*r* = −0.521, *P* < 0.001), followed by E/e’/LASr and METs (*r* = −0.462, *P* < 0.001).

**Table 4:** Multivariate correlation analysis and optimization model prediction.

Clinical model 1	*β*	SE	Stand-B	*t*	*P*
Age	−0.220	0.011	−0.115	−1.741	**0.023**
HR-rest	0.010	0.010	0.065	0.991	0.323
BMI	−0.916	0.040	−0.196	−2.925	**0.004**
BSA	−0.449	0.609	−0.080	−1.229	0.221
SBP-rest	−0.174	0.006	−0.143	−2.189	**0.032**
Echocardiographic model 2a					
E/e’/LASr	−2.266	0.661	−0.235	−3.427	**0.001**
LAVI	−0.045	0.016	−0.180	−2.715	**0.007**
GCW	0.200	0.000	0.032	1.421	0.157
GLS	−0.010	0.028	−0.024	−0.357	0.721
SVRI	0.100	0.000	0.072	1.125	0.262
RVSP	−0.074	0.024	−0.209	−1.91	**0.027**
E/e’	−0.215	0.064	−0.319	−3.152	0.003
Model 2					
E/e’/LASr-c	−1.390	0.577	−0.173	−2.907	**0.001**
LAVI	−0.150	0.017	−0.199	−2.680	**0.003**
GCW	0.012	0.000	0.042	1.459	0.146
GLS	−0.008	0.028	−0.020	−0.299	0.765
SVRI	0.010	0.000	0.075	1.159	0.248
RVSP	−0.667	0.024	−0.189	−2.450	**0.003**
E/e’	−0.145	0.062	−0.216	−2.148	**0.02**
Model 3 All					
LAVI	−0.040	0.016	−0.161	−1.846	0.055
RVSP	−0.248	0.025	−0.138	−2.237	**0.014**
Age	−0.022	0.028	−0.053	−0.791	0.430
BMI	−0.109	0.037	−0.082	−2.905	0.723
SBP-rest	−0.004	0.007	−0.045	−0.652	0.515
E/e’	−0.192	0.073	−0.285	−2.315	**0.012**
E/e’/LASr	−1.143	0.280	−0.218	−2.91	**0.006**
E/e’/LASr-c	−0.780	0.794	−0.185	−2.242	**0.058**

HT, hypertension; HFpEF, heart failure with preserved ejection fraction; statistically significant *P* values are shown.

Values with *P* < 0.05 are highlighted in bold.

### Analysis of the LASI to identify HT-HFpEF with reduced exercise capacity

**Figure 6:**
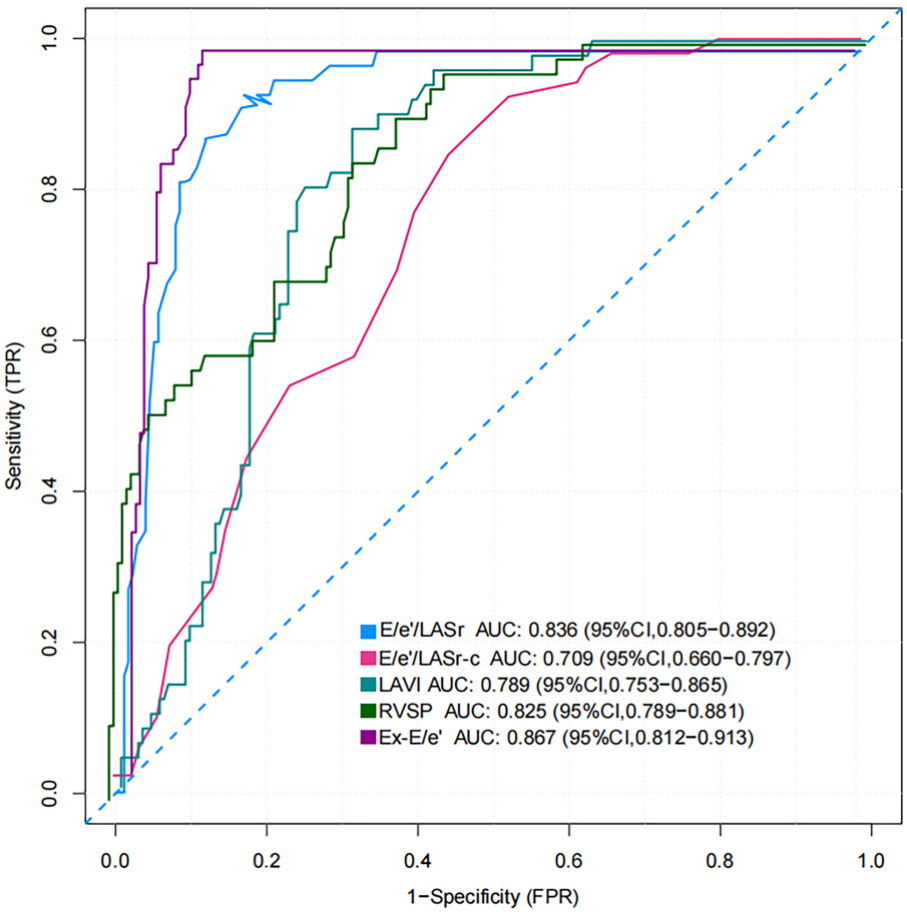
ROC curve and AUC value for identify HT-HFpEF with reduced exercise capacity.

The AUC and 95% CI values for the variables are presented in Figure 6. The following cut-off values and AUCs were identified: E/e'/LASr (cut-off: 0.46; AUC: 0.836; 95% CI: 0.805–0.892), and E/e'/LASr-c (cut-off: 0.33; AUC: 0.709; 95% CI: 0.660–0.797). Additionally, the diagnostic performance for LAVI (AUC: 0.789; 95% CI: 0.753–0.865), Ex-E/e' (AUC: 0.867 95% CI: 0.812–0.913), and RVSP (AUC: 0.825; 95% CI: 0.789–0.881) were evaluated. No significant differences were found between E/e'/LASr and Ex-E/e' (Delong test, *P* = 0.239) (Graphical Abstract).

## Discussion

In recent years, strain analysis conducted across various time periods has emerged as precise method for assessing crucial atrial function-related parameters (17), with LASI standing out as a novel and highly effective metric (18). The rapid acquisition and analysis of 3D LA images provide valuable insights into reservoir,conduit and active contraction function, serving as pivotal force in promoting blood flow into the ventricle (19). Given that a significant proportion of HFpEF patients possess prior history of hypertension (20), we conducted distinctive investigation focusing on this subset, comparing LA functional traits between individuals with HT and those suffering from HT-HFpEF. The identification of HT-HFpEF patients with reduced exercise tolerance is crucial for guiding targeted interventions and preventing progression to irreversible HFpEF (21). The key findings identified LASI analysis can be utilized as functional biomarker for evaluating HT and HT-HFpEF patients. And multiple regression analyses revealed E/e'/LASr remained an independent factor associated with exercise capacity.

There is currently no conclusive evidence regarding the association between LA function and LV mechanical abnormalities in the progression of exercise intolerance among patients with HT-HFpEF. This is primarily due to the potential coexistence or disproportionate involvement of LA and LV cardiomyopathy. Understanding the various stages of hypertensive heart disease evolution is crucial for elucidating the progression of HT-HFpEF, based on pathophysiological mechanisms (22, 23). A separate study (24) has reported that LASI is linked to the progression of HFpEF and may serve as predictor of invasive hemodynamic abnormalities.In this study, patients with the HT-HFpEF subtype were screened to exclude significant confounding factors, enabling more focused investigation of LA function changes and minimizing the influence of inherent atrial dysfunction typically observed in atrial fibrillation (25–27). This subtype is likely more associated with myocardial hypertrophy and fibrosis, leading to left ventricular diastolic dysfunction and elevated left atrial pressure (28). It differs from HFpEF related to metabolic syndrome, pulmonary artery disease, highlighting the significance of our study in investigating this as a distinct subtype (29). Comparative analysis revealed no significant differences in Ea/Ees among the three groups,however, LA volume and strain were found to be impaired to varying degrees in patients with HT, suggesting that atrial dysfunction may precede the impairment of pressure-volume loop function (30).

The 3D left atrial assessment technique has been reported in previous studies and has been validated as a robust tool for evaluating left atrial function (31), myocardial deformation and arterial stiffness can detect and predict subclinical cardiotoxicity in patients with anthracyclines-induced cardiotoxicity (32, 33). The LASr, LASr-c, LASct-c differed significantly between the HT and HT-HFpEF group, which suggested that these variables serve as sensitive indicators of cardiac dysfunction, and additional research and validation are warranted in this domain (34, 35). Previous studies (36–38) have documented the association between LA function and exercise tolerance. Abnormalities in myocyte relaxation and progressive extracellular fibrosis can result in compromised ventricular filling, a process influenced by both pressure overload and neuro-endocrine factors, ultimately culminating in elevated LA pressure and dyspnea (39–41). Furthermore, multiple regression analysis model identified E/e'/LASr as an independent predictor of METs, indicating that progressive LA remodeling contributes to decreased atrioventricular compliance and is directly associated with exercise intolerance. Additionally, circumferential reserve strain also exhibited strong correlation with METs.

Kim D et al. (42) demonstrated that 2D speckle-tracking-based LASI is associated with increased all-cause mortality and higher risk of hospitalization in HFpEF patients, with its prognostic value exceeding that of LV filling pressure indices.In the present study, LASI was employed to investigate patients with HT-HFpEF and exercise intolerance,the results revealed a consistent trend in LASI (E/e'/LASr, E/e'/LASr-c), with further elevation observed in the HT-HFpEF group, highlighting its significance as key indicator of impaired atrioventricular compliance. Consequently, thorough assessment of atrioventricular compliance, combined with exercise tolerance and LA reservoir strain, may hold significant incremental value in differentiating subgroups of HT-HFpEF patients, ultimately leading to more tailored and personalized therapeutic interventions.

### Limitations

In this study, we mainly focused on the hypertensive subtype of HFpEF, However,the sample size was relatively small and needs to be increased in future work. For the heterogeneity of HFpEF, including patients both with hypertension and renal dysfunction, did not entirely excluded. Despite the use of high-quality spatial and temporal resolution in our three-dimensional speckle tracking analysis, there was still inter-observer difference,and intervendor variability, image quality on loading conditions that could have theoretically impacted upon our results. In addition, long-term follow-up and large-scale prospective studies are now needed to determine the clinical predictive value of early changes in left atrial function in patients with HT.Furthermore, more in-depth analysis of pathological changes in the left atrium is required across different cardiovascular diseases to enhance our understanding of the relevant mechanisms involved. In addition, LVEDP, E/e',and LASr may be affected by volume overload and may change with the use of diuretics. We lacked objective data relating to the assessment of pulmonary capillary wedge pressure (PCWP) based on invasive hemodynamics. Further studies are needed to investigate whethe increased LASI can represent an independent determinant for predicting HF hospitalization or mortality.

## Conclusions

Progressive LA remodeling contributes to decreased atrioventricular compliance in HT and HT-HFpEF patients, and E/e'/LASr is an independent indicator of exercise capacity and can identify HT-HFpEF patients with exercise intolerance. Furthermore, the emerging metric of 3D strain offers valuable additional insights into the evaluation of atrial function in the HFpEF population.

## Data Availability

The raw data supporting the conclusions of this article will be made available by the authors, without undue reservation.
